# Supplementary material for: Solar-blind ultraviolet-C persistent luminescence phosphors
Source: Nat Commun. 2020 Apr 27;11:2040. doi: 10.1038/s41467-020-16015-z (PMC7184723; doi:10.1038/s41467-020-16015-z)
Supplement: Supplementary file 1 — Supplementary Information [file 41467_2020_16015_MOESM1_ESM.pdf]

Supplementary Information

Solar-blind ultraviolet-C persistent luminescence phosphors

Wang *et al.*

Supplementary Note 1. Estimation and determination of persistent luminescence power intensity of $Ca_2Al_2SiO_7:Pr^{3+}$ persistent phosphor

We used a Newport 2936-R optical power and energy meter and a Newport 918D-UV-OD3R UV enhanced silicon photodetector to measure the persistent luminescence intensities of a $Ca_2Al_2SiO_7:Pr^{3+}$ persistent phosphor disc at different decay instants in the initial decay period of 10 s to 120 s. The measurement system has a minimum measurable power of 20 pW. Based on the measured intensities, we used a semi-sphere irradiation geometry model to estimate and determine the UVC persistent luminescence power intensities of the $Ca_2Al_2SiO_7:Pr^{3+}$ disc in various initial decay instants, e.g., 1 s, 10 s, 30 s and 60 s, in the absolute unit of mW m^{-2}.

Supplementary Figure 2a shows the experimental setup for the measurement of persistent luminescence intensities of a $Ca_2Al_2SiO_7:Pr^{3+}$ disc. A carton (size: L × W × H = 24 × 22 × 16.5 mm) was used as a small dark chamber. The UV silicon photodetector was adhered onto the bottom of the carton with the sensor facing upward. The diameter of the sensor area is 10 mm. A hole with diameter of 14 mm was made on the top board of the carton with its center aligned with the sensor center. The distance between the surface of the sensor and the surface of the top board is 150 mm. A $Ca_2Al_2SiO_7:Pr^{3+}$ disc with diameter of 15 mm was irradiated using a 4 W 254 nm UV lamp for 2 min. After the lamp was ceased, the charged disc was immediately placed on the hole and the readout on the Newport 2936-R meter was recorded from 10 s after the excitation was ceased and last to 120 s in 10 s steps, as shown in Supplementary Table 1. During the excitation and measurement, the light in the lab was turned off, making the measurement inside the carton chamber in a completely dark condition.

The grey dash curve in Supplementary Fig. 2b is the decay curve of $Ca_2Al_2SiO_7:Pr^{3+}$ persistent phosphor in the initial decay period (10–300 s) monitored at 268 nm emission. We normalized the decay curve at the 10 s decay instant and obtained the relationship between the persistent luminescence intensity (I) and the decay time (t), i.e.,

$$I = 0.03286 + 2.08578\exp(-t/8.81733) + 0.35426\exp(-t/54.67284)$$

The red curve in Supplementary Fig. 2b is the fitting relationship of I–t. The measured persistent luminescence intensities were also normalized at 10 s and they fit the I-t plot very well, as shown by the green dots in Supplementary Fig. 2b.

According to the fitting I-t relationship, the intensity of persistent luminescence at t = 1 s is 2.24 times of that at 10 s. Therefore, basing on Supplementary Table 1, we deduced the power measured by the UV photodetector at t = 1 s instant would be 2.03 nW.

Supplementary Figure 2c shows the persistent luminescence emission spectrum of $Ca_2Al_2SiO_7:Pr^{3+}$ persistent phosphor acquired at 10 s decay. Besides UVC emission at 250–280 nm (region I), the spectrum also contains emission in the 280–400 nm range (region II). We integrated the areas of region I and region II and found that region I accounted for 45.7% of the whole emission area. Therefore, to calculate the UVC persistent luminescence power intensity, the intensity measured by the UV photodetector needs to be multiplied by 0.457.

Finally, we assumed the UVC persistent luminescence at the position of disc was semi-sphere irradiation and the intensity at each position on the disc was the same as that at the center position. We calculated the UVC persistent luminescence intensity at the disc position using equation, $I = 0.457 \times I_m \times 2\pi \times h^2/(\pi \times r^2)$, where I_m is the measured intensity by the power meter (Supplementary Table 1), h is the distance between the surface of the sensor and the surface of the top board, which is 150 mm, and r is the radius of sensor, which is 5 mm. The UVC persistent luminescence power intensity was then obtained by dividing the intensity (I) with the area of the hole ($= \pi \times (7\ \text{mm})^2 = 153.94\ \text{mm}^2$).

Based on the above analysis and the data in Supplementary Table 1, the UVC persistent luminescence power intensities of $Ca_2Al_2SiO_7:Pr^{3+}$ persistent phosphor at 1 s, 10 s, 30 s and 60 s decays are calculated to be about 10.9 mW m^{-2}, 4.8 mW m^{-2}, 1.6 mW m^{-2} and 0.8 mW m^{-2}, respectively.

Supplementary Table 1. Intensities measured by a Newport UV photodetector (Source data are provided as a Source Data file).

Decay time (s)	Intensity, I_m (pW)
10	905.6
20	402.5
30	296.0
40	220.4
50	155.1
60	142.6
70	115.1
80	103.2
90	92.7
100	86.7
110	79.9
120	71.3

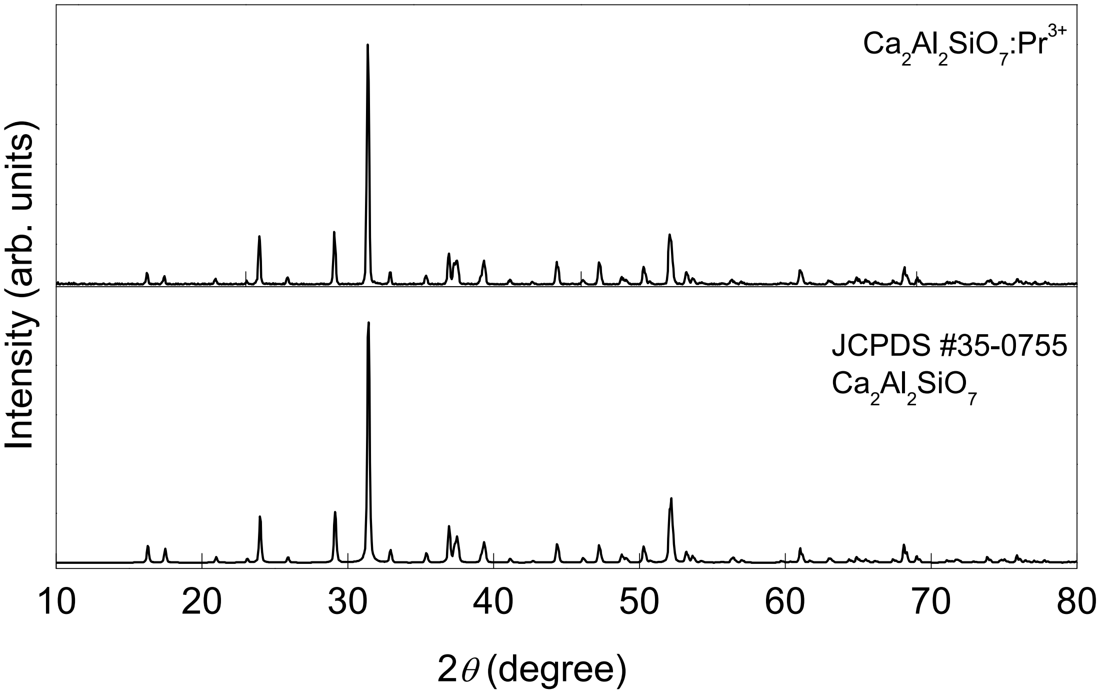

Supplementary Figure 1. X-ray diffraction pattern of $Ca_2Al_2SiO_7$:Pr^{3+} persistent phosphor. The $Ca_2Al_2SiO_7$:Pr^{3+} phosphor has the melilite structure. Standard data of gehlenite $Ca_2Al_2SiO_7$ (JCPDS #35-0755) is also displayed as reference. Gehlenite is an Al-rich member of the melilite group. Source data are provided as a Source Data file.

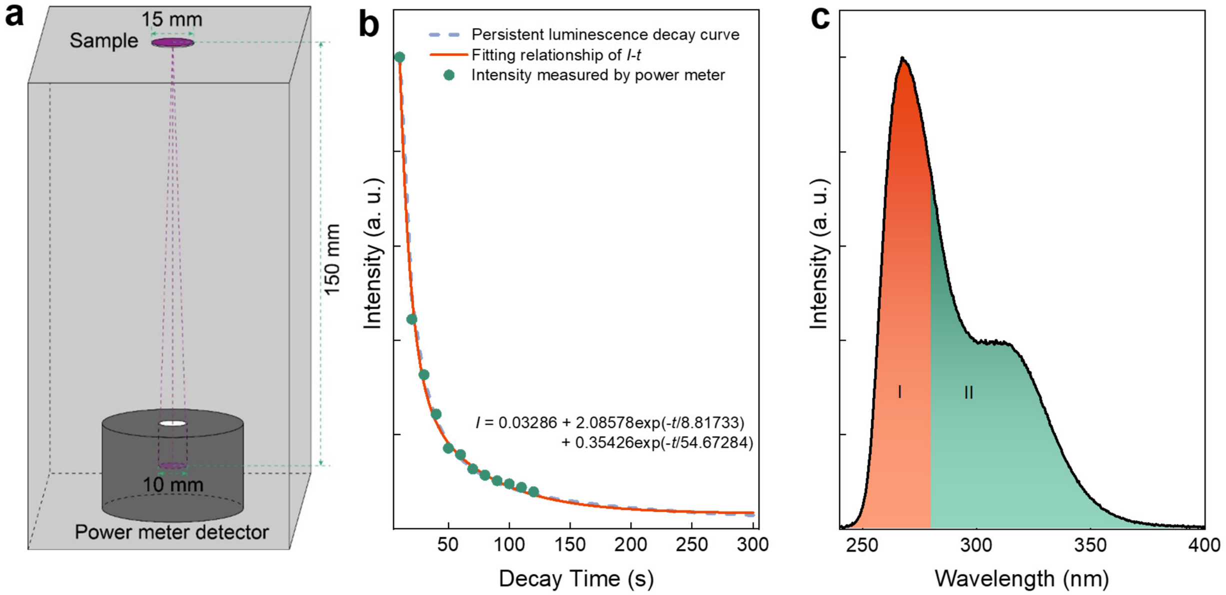

Supplementary Figure 2. Estimation of UVC persistent luminescence power intensities of a $Ca_2Al_2SiO_7$:Pr^{3+} disc. (**a**) Measurement setup. The measurement was carried out in a carton dark chamber. A Newport 918D-UV-OD3R UV enhanced silicon photodetector was adhered onto the bottom of the carton with the sensor facing upward. The diameter of the sensor area is 10 mm. A hole with diameter of 14 mm was made on the top board of the carton with its center aligned with the sensor center. The distance between the surface of the sensor and the surface of the top board is 150 mm. The diameter of the disc sample is 15 mm. For the measurement, the disc was irradiated by a 254 nm lamp for 2 min and was then immediately placed on top of the hole. The measured persistent luminescence intensities are shown in Supplementary Table 1. (**b**) Persistent luminescence decay curve (grey dash curve) monitored at 268 nm. The decay curve was normalized at the 10 s decay instant and the red line curve is the thus obtained fitting relationship between the persistent luminescence intensity (I) and the decay time (t). The measured persistent luminescence intensities were also normalized at the 10 s decay instant and were displayed as green dots in the figure. The measured intensities fit the I–t plot very well. (**c**) Persistent luminescence emission spectrum acquired at 10 s decay. Region I represents the UVC portion of the persistent luminescence, which accounts for 45.7% of the whole emission area and was used to calculated the UVC persistent luminescence power intensity. Source data for (**b**) and (**c**) are provided as a Source Data file.

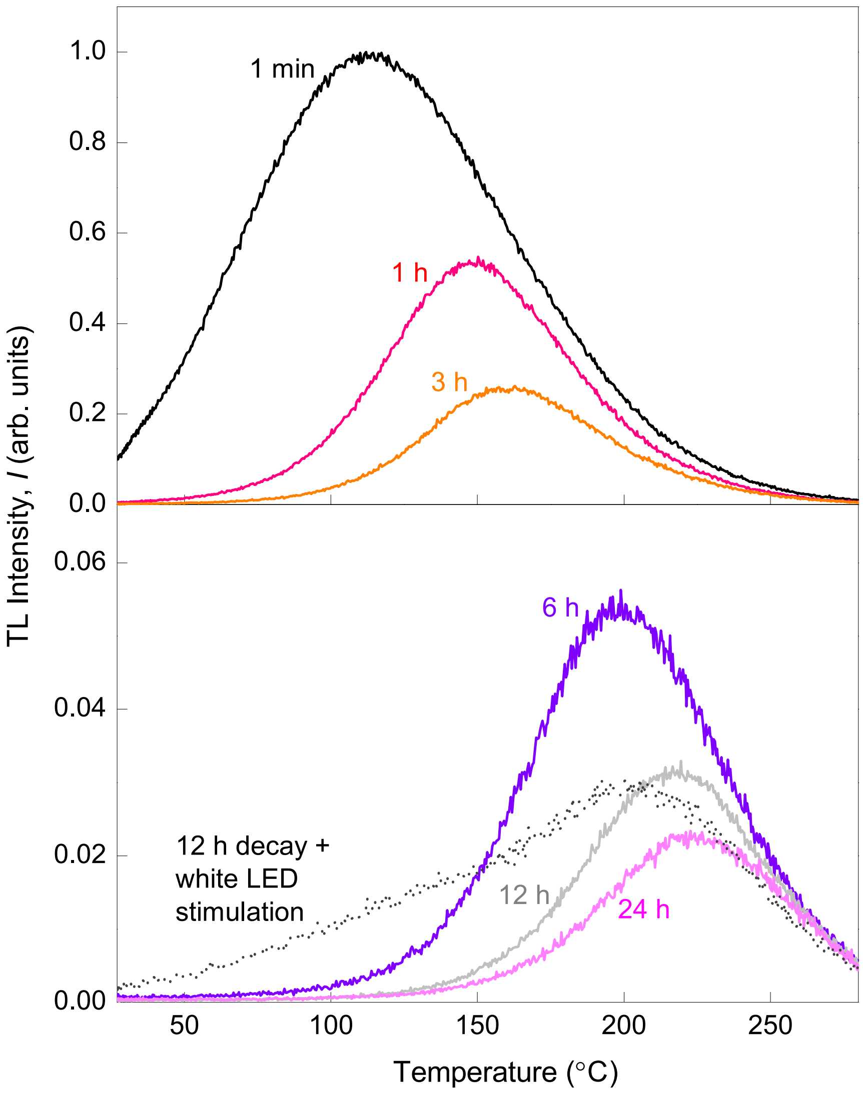

Supplementary Figure 3. Thermoluminescence curves of $Ca_2Al_2SiO_7$:Pr^{3+} persistent phosphor discs. The discs were pre-irradiated by a 254 nm lamp for 2 min and underwent 1 min to 24 h decay in darkness before measurement. The discs were heated from 25° to 280 °C with a heating rate of 4 °C s^{-1}. The monitoring wavelength is 268 nm. The black dots-line curve in the bottom panel was acquired on a 12 h decayed disc after stimulation by a white LED flashlight for 20 s. Compared with the 12 h decayed disc but without stimulation (grey curve), the LED flashlight stimulation causes the redistribution of electrons in the energy traps; that is, some electrons are transferred from the deep traps to the shallow traps, resulting in enhanced persistent luminescence signal, i.e., PSPL signal, as shown in Figure 4 of the main text. Source data are provided as a Source Data file.

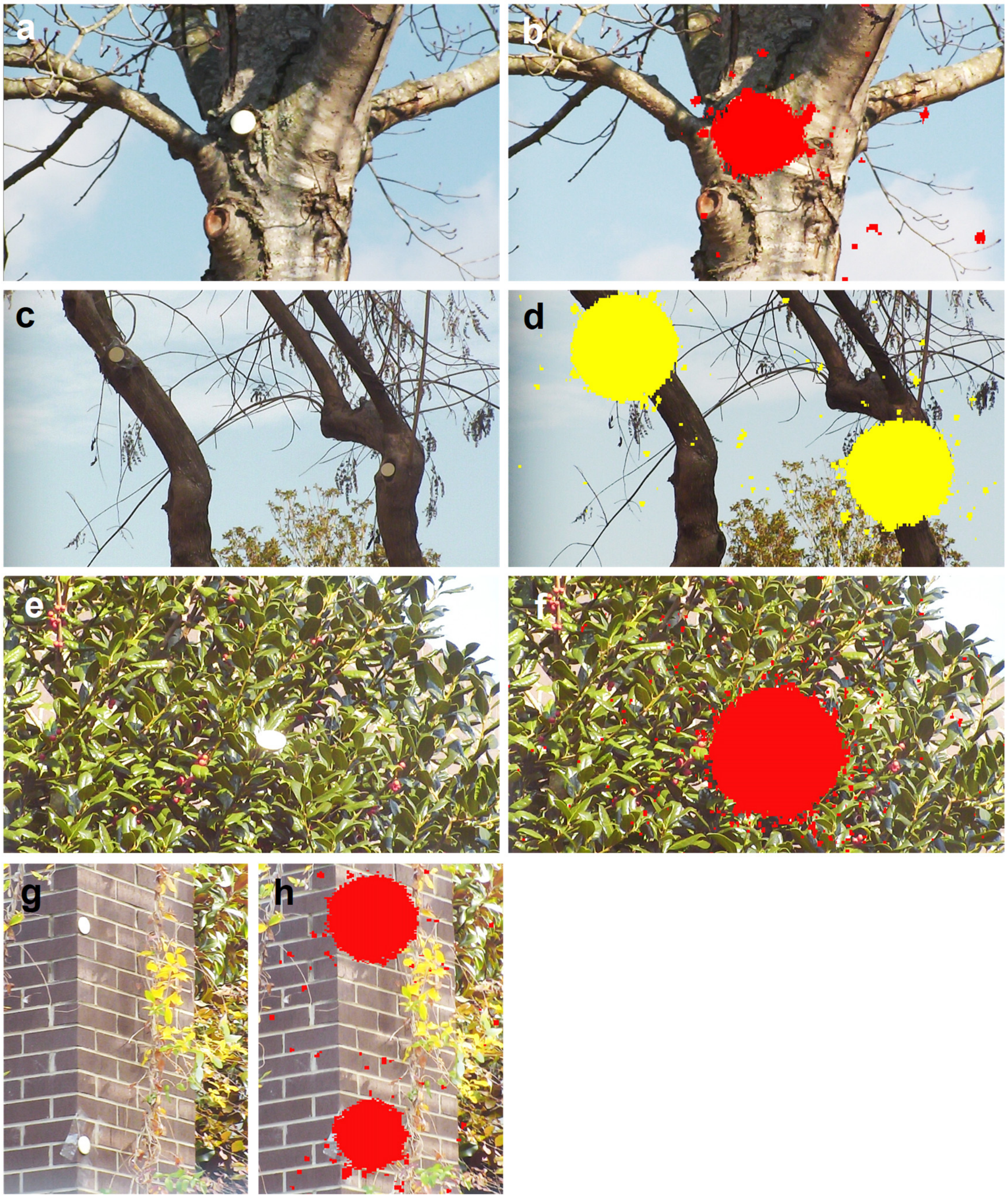

Supplementary Figure 4. UVC radiation images of 50 mm diameter $Ca_2Al_2SiO_7:Pr^{3+}$ discs placed in various outdoor locations in direct sunlight. (**a**,**b**) One disc was adhered on a tree trunk. **(c**,**d**) Two discs were adhered on two tree trunks. (**e**,**f**) One disc was placed in bush. (**g**,**h**) Two discs were adhered on a brick wall. In these imaging experiments, the samples were irradiated by a 254 nm lamp for 2 min. The UVC images were taken by a corona camera at about 2 min after ceasing the lamp. The camera was located about 10 m for (**a**–**d**) and 5 m for (**e**–**h**) away from the samples. The UVC radiation is represented by red or yellow color.

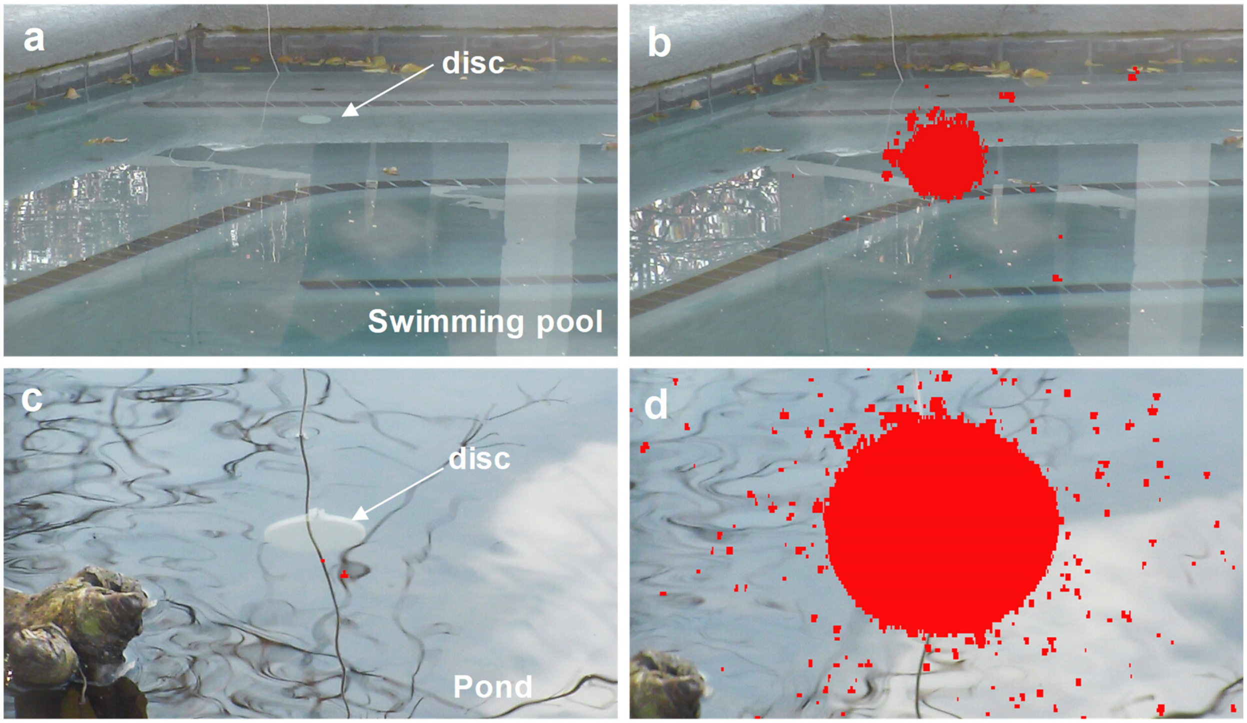

Supplementary Figure 5. UVC radiation images of 50 mm diameter $Ca_2Al_2SiO_7:Pr^{3+}$ discs immersed in water in direct sunlight. (**a**,**b**) A disc immersed in a swimming pool at about 10 cm depth. (**c**,**d**) A disc immersed in a pond at about 5 cm depth. Before immersing in water, the samples were irradiated by a battery-powered 4-W 254 nm lamp for 2 min. The charged discs were then immersed into water and the images were taken by a corona camera at about 2 min after ceasing the lamp. The distance between the sample and camera is about 10 m for in swimming pool and 3 m for in pond. The UVC radiation is represented by red color.

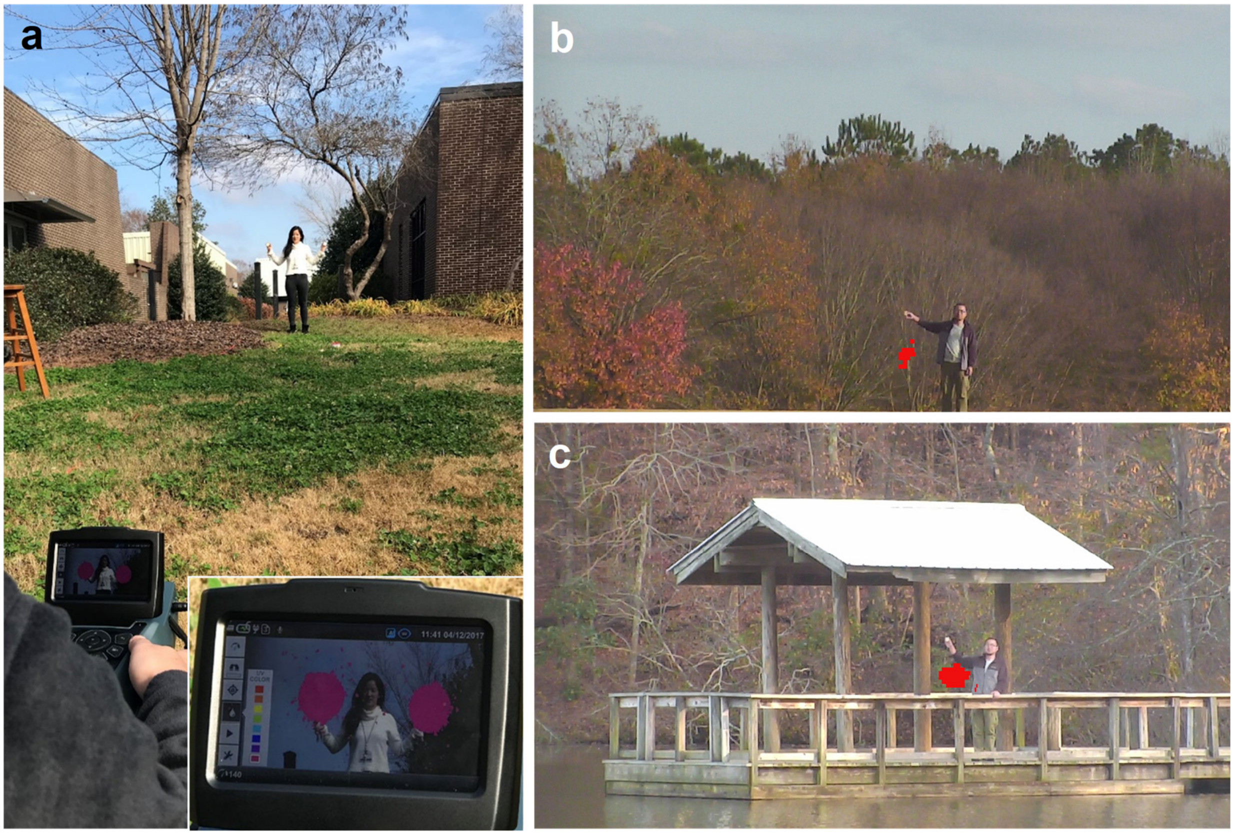

Supplementary Figure 6. Imaging of UVC radiation of 50 mm diameter $Ca_2Al_2SiO_7:Pr^{3+}$ discs from long distance in direct sunlight. (**a**) A cell phone image showing the screen of a corona camera that displays the UVC radiation of two glowing discs held by a person. The distance between the discs and camera is about 20 m. Inset is an enlarged image of the camera screen. The discs were irradiated by a 254 nm lamp for 2 min and the UVC radiation was imaged at about 2 min after ceasing the lamp. (**b**) A glowing disc held by a person from about 50 m away in an open area. (**c**) A glowing disc held by a person standing on a wood trestle bridge in a lake from about 50 m away. In (**b**) and (**c**), the discs were irradiated by a battery-powered 4-W 254 nm lamp for 2 min and the UVC radiation was imaged at about 2 min after ceasing the lamp. Also in (**b**) and (**c**), the UVC radiation spot (in red) does not overlay onto the disc (in the visible image), because the focal planes of the UVC sensor and visible sensor are not precisely overlaid. In these imaging experiments, care was taken to avoid the operators from being exposed to the UVC radiation from the glowing samples.

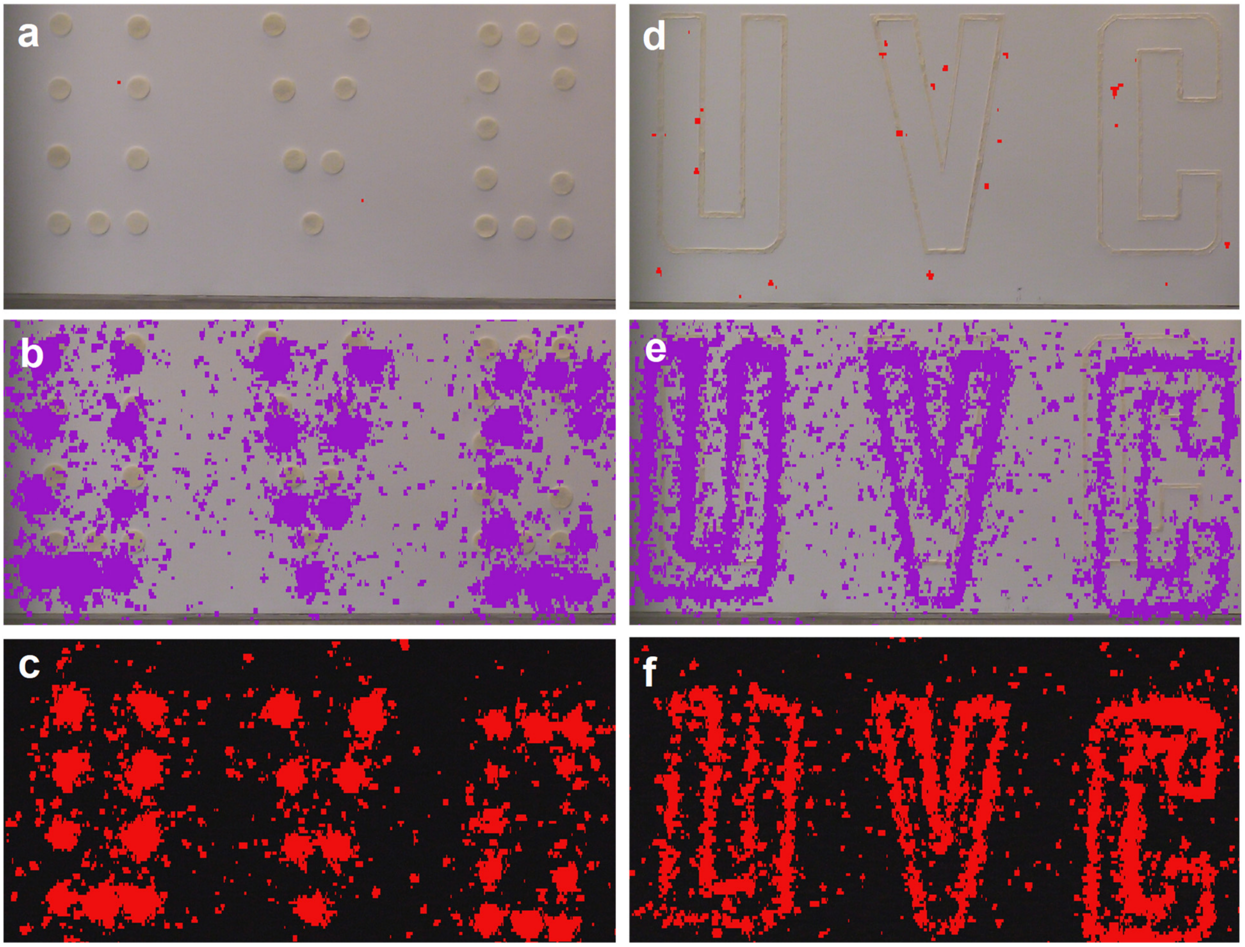

Supplementary Figure 7. UVC radiation images of $Ca_2Al_2SiO_7$:Pr^{3+} discs and paint taken by a corona camera in room light and in darkness. (**a**) Visible image of letters “U”, “V” and “C” made of 20 mm diameter discs on a white wood board. The disc letters were then irradiated by a 254 nm lamp for 2 min. (**b**) UVC image of the disc letters taken at 2 min decay in room light. (**c**) UVC image of the disc letters taken at 2 min decay in darkness. (**d**) Visible image of hollow letters “U”, “V” and “C” written using UVC paint on a white wood board. The paint letters were then irradiated by a 254 nm lamp for 2 min. (**e**) UVC image of the three paint letters taken at 2 min decay in room light. (**f**) UVC image of the three paint letters taken at 2 min decay in darkness. For in room light, the UVC radiation is represented by purple color; for in darkness, the UVC radiation is represented by red color.

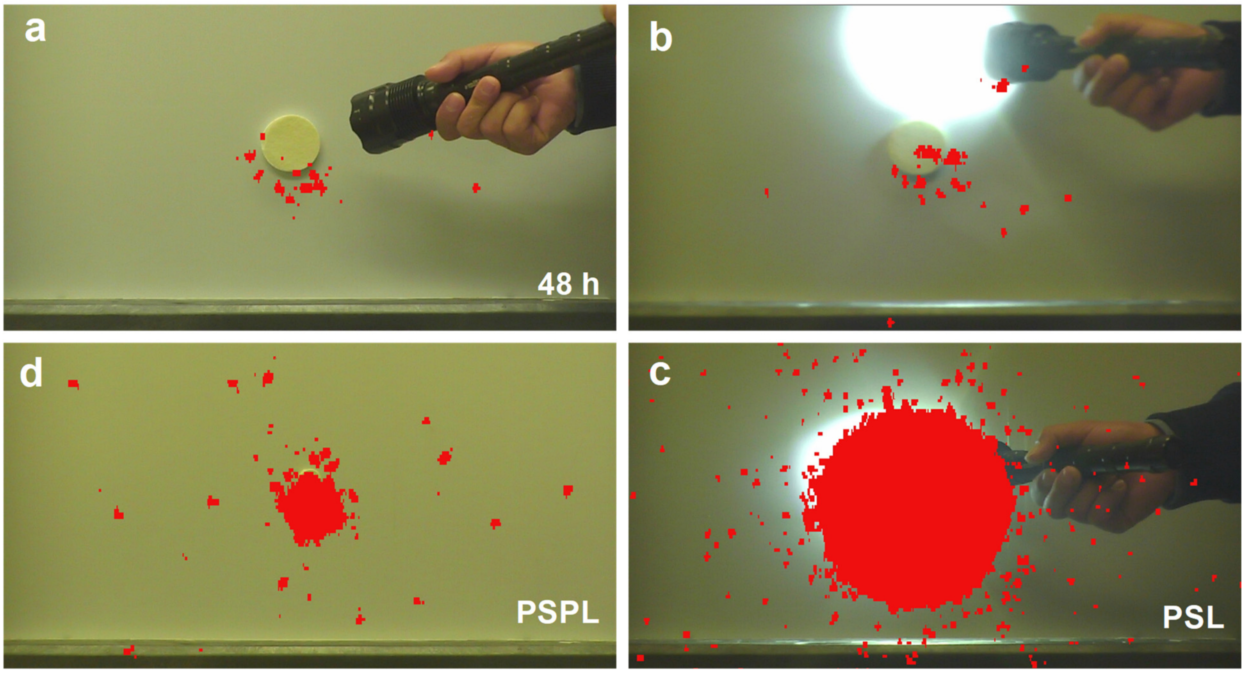

Supplementary Figure 8. UVC photostimulated luminescence (PSL) and photostimulated persistent luminescence (PSPL) images of a 50 mm diameter $Ca_2Al_2SiO_7:Pr^{3+}$ disc taken by a corona camera in room light. (**a**) A 48 h decayed disc (in room light) showing very weak UVC afterglow. The disc was adhered on a white wood board (the image appears in light yellow due to the lighting issue). (**b**) A 3800 Lumen white LED flashlight was irradiating on the board. No enhanced UVC radiation was observed. (**c**) The white LED flashlight was irradiating on the decayed disc. Strong UVC PSL signal was obtained. (**d**) After ceasing the flashlight stimulation (for 10 s), the disc exhibited enhanced UVC persistent luminescence signal, showing the PSPL effect. The image was taken at 1 min after ceasing the flashlight.

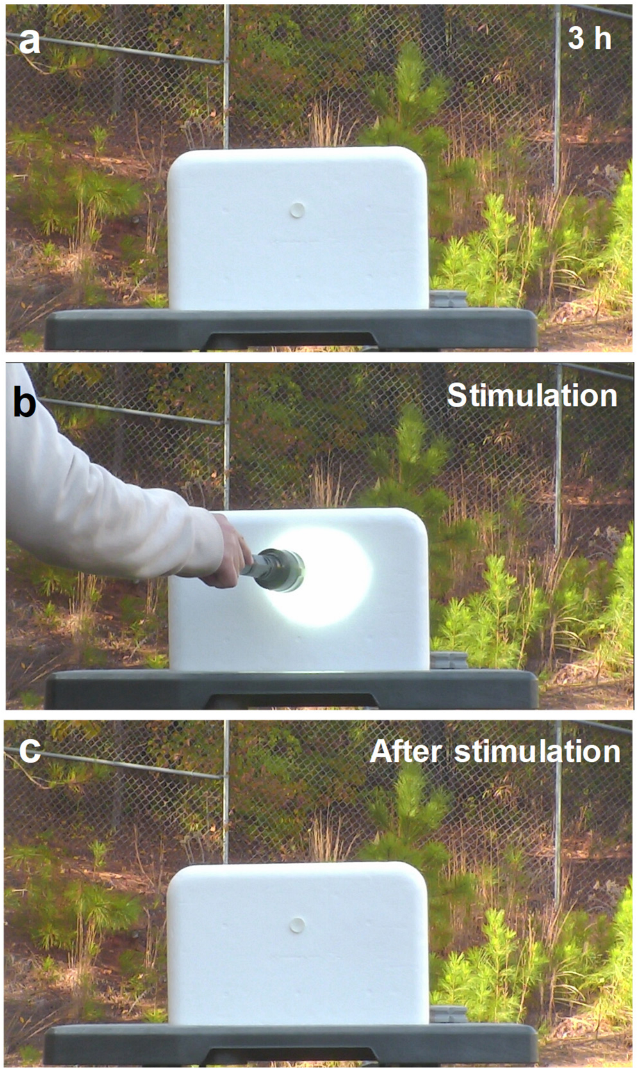

Supplementary Figure 9. Photostimulation experiment on a 3 h decayed 20 mm diameter $Ca_2Al_2SiO_7$:Pr^{3+} disc in direct sunlight. (**a**) After decay in direct sunlight for 3 h, no UVC signal was detected from the disc by a corona camera. (**b**) A 3800 Lumen white LED flashlight was irradiating on the disc. No UVC PSL signal was observed. (**c**) After ceasing the flashlight irradiation, no UVC PSPL signal was observed. The results show that the disc was completely bleached (i.e., all energy traps in the disc were emptied) after decay in indirect sunlight for 3 h.

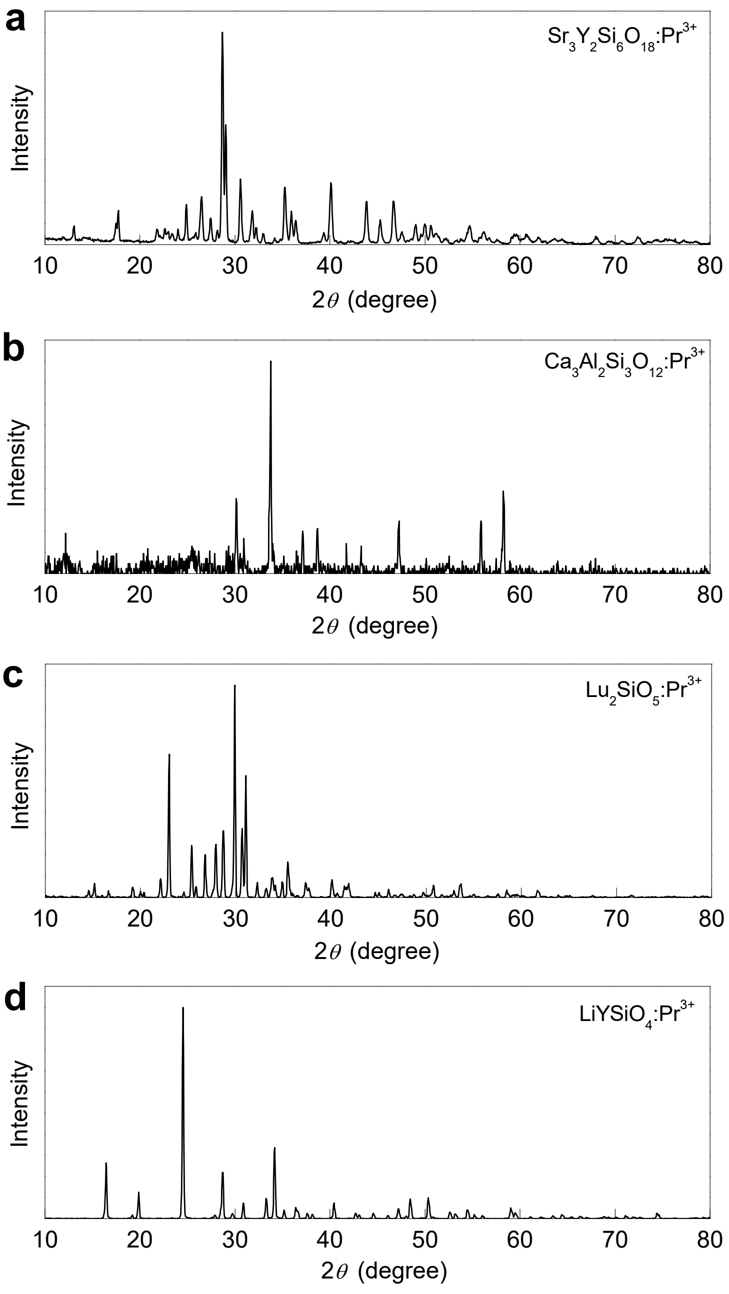

Supplementary Figure 10. X-ray diffraction patterns of $Sr_3Y_2Si_6O_{18}:Pr^{3+}$, $Ca_3Al_2Si_3O_{12}:Pr^{3+}$, $Lu_2SiO_5:Pr^{3+}$ and $LiYSiO_4:Pr^{3+}$ phosphors. The pattern for $Sr_3Y_2Si_6O_{18}:Pr^{3+}$ can be indexed as a cyclosilicate structure according to the pattern of $Sr_3Er_2Si_6O_{18}$ (see main text). The pattern for $Ca_3Al_2Si_3O_{12}:Pr^{3+}$ can be indexed as a grossular garnet structure according to JCPDS #39-0368. The pattern for $Lu_2SiO_5:Pr^{3+}$ can be indexed as an oxyorthosilicate structure according to JCPDS #41-239. The pattern for $LiYSiO_4:Pr^{3+}$ can be indexed as an orthosilicate structure according to JCPDS #82-1449. Source data are provided as a Source Data file.

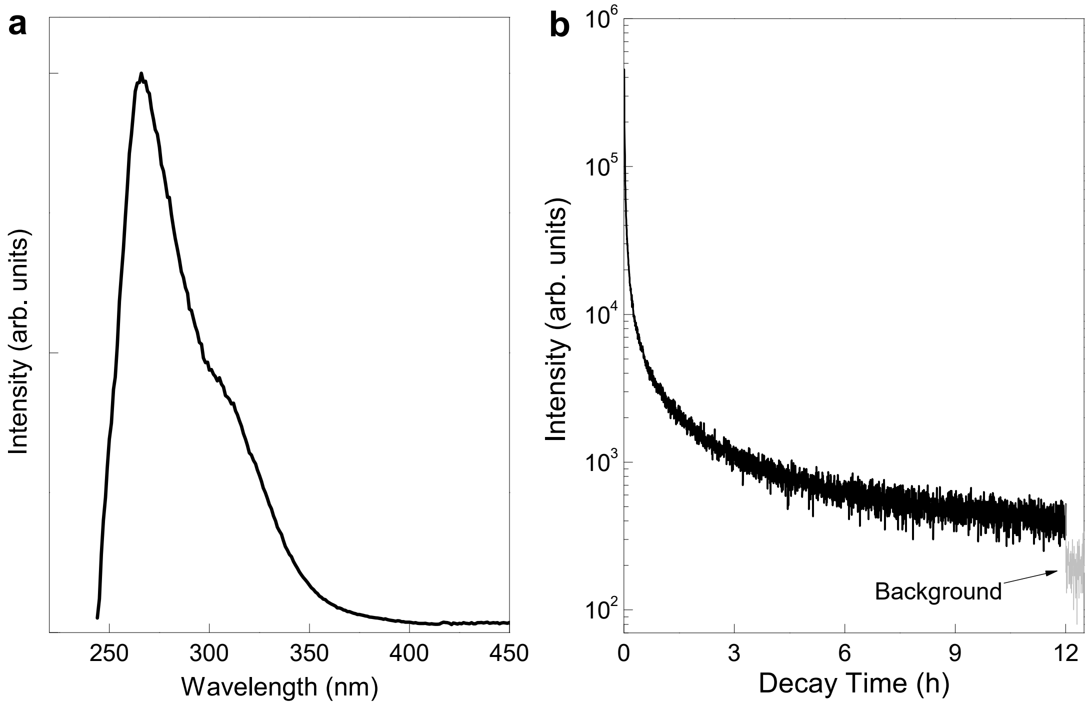

Supplementary Figure 11. Photoluminescence and persistent luminescence of cyclosilicate $Sr_3Y_2Si_6O_{18}:Pr^{3+}$ UVC persistent phosphor discs at room temperature. (**a**) Photoluminescence emission spectrum under 220 nm light excitation. The emission band peaks at 266 nm. (**b**) UVC persistent luminescence decay curve monitored at 265 nm emission. The disc was pre-irradiated by a 254 nm lamp for 2 min and the decay measurement lasted for 12 h. Source data are provided as a Source Data file.

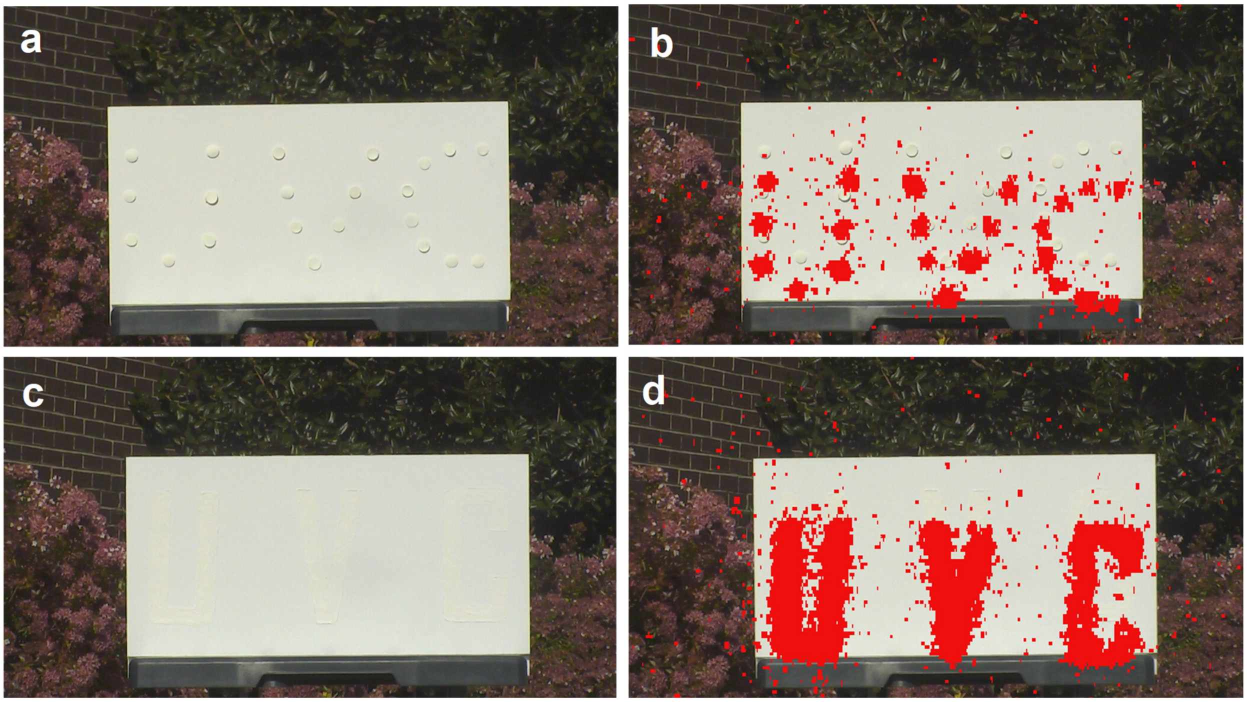

Supplementary Figure 12. UVC radiation images of $Sr_3Y_2Si_6O_{18}:Pr^{3+}$ discs and paint taken by a corona camera in direct sunlight. (**a**) Visible image of letters "U", "V" and "C" made of 20 mm diameter discs on a white wood board. (**b**) UVC image of the disc letters taken at 2 min decay. (**c**) Visible image of solid letters "U", "V" and "C" written using UVC paint on a white wood board. (**d**) UVC image of the paint letters taken at 2 min decay. In these imaging experiments, the samples were irradiated by a 254 nm lamp for 2 min. The camera was located about 8 m away from the samples.

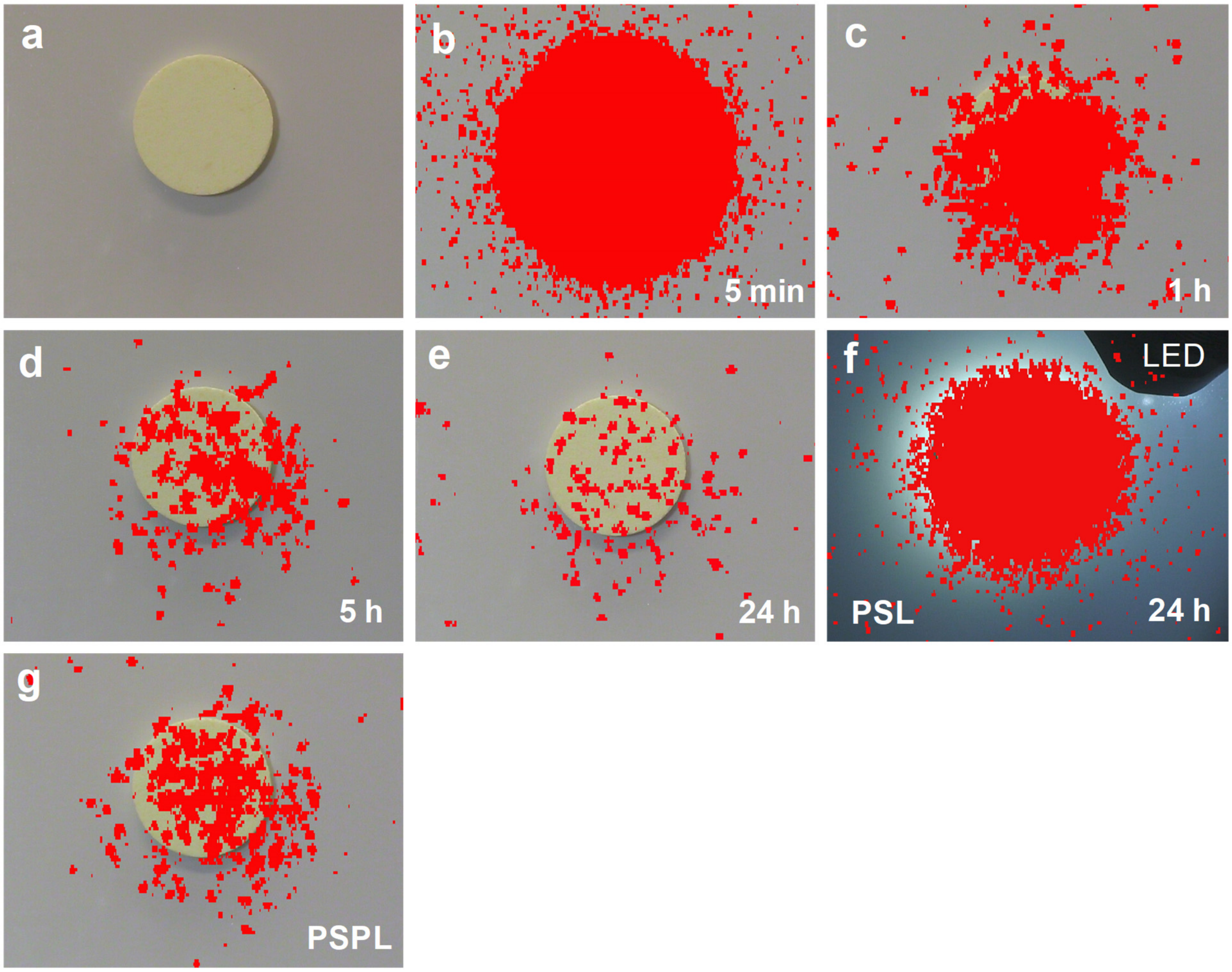

Supplementary Figure 13. UVC persistent luminescence, PSL and PSPL images of a 50 mm diameter $Sr_3Y_2Si_6O_{18}:Pr^{3+}$ disc taken by a corona camera in room light. (**a**) Visible image of the disc. The disc was then irradiated by a 254 nm lamp for 2 min. (**b**–**e**) UVC images of the disc taken at different decay time (5 min to 24 h). (**f**) UVC PSL image of the 24 h decayed disc being irradiated by a 3800 Lumen white LED flashlight. The irradiation lasted for 10 s. (**g**) UVC PSPL image taken at 1 min after ceasing the flashlight irradiation.

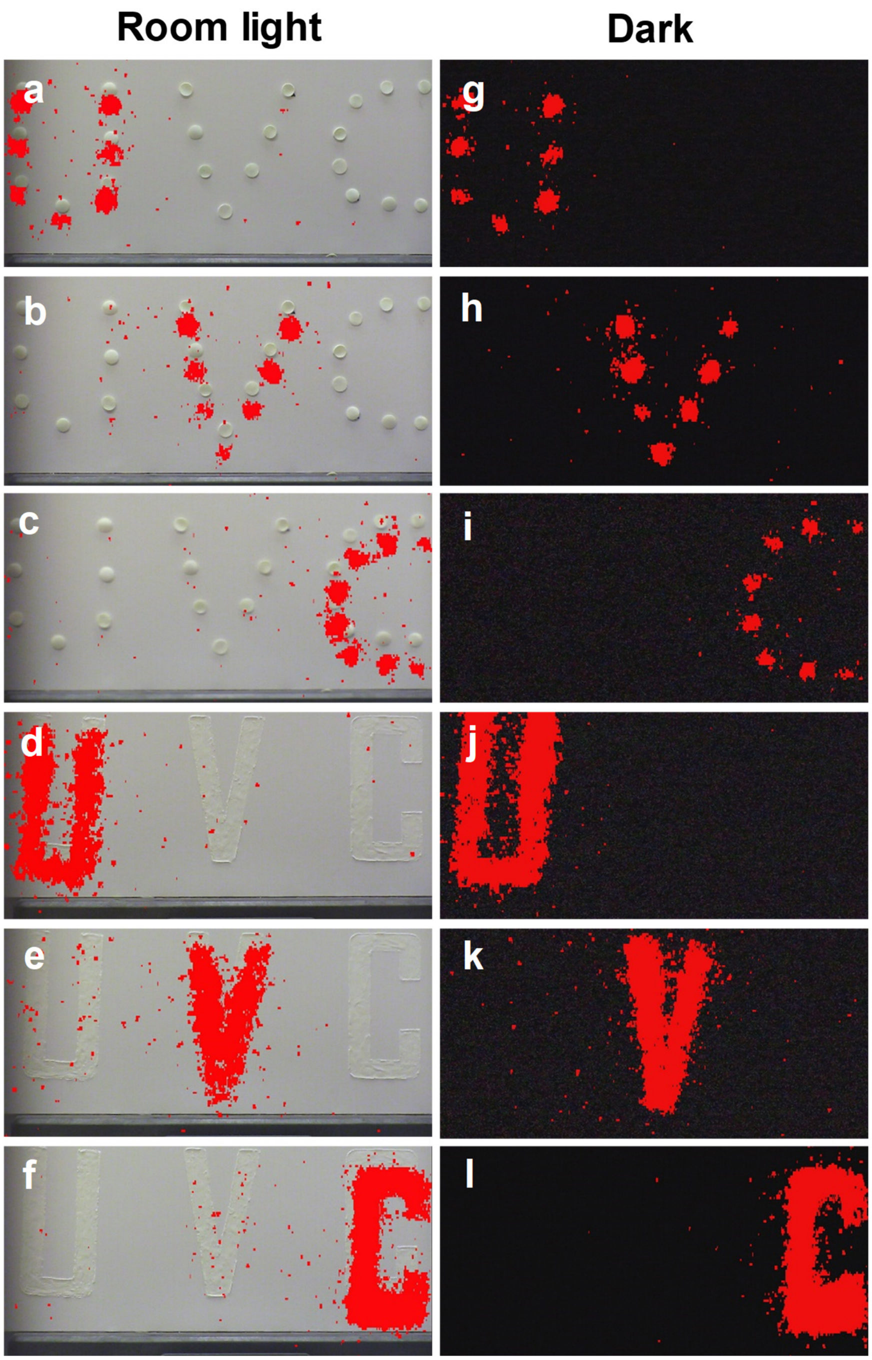

Supplementary Figure 14. UVC radiation images of $Sr_3Y_2Si_6O_{18}$:Pr^{3+} discs and paint taken by a corona camera in room light and in darkness. (**a–c**) UVC images of letters “U”, “V” and “C” made of 20 mm diameter discs in room light. (**d–f**) UVC images of letters “U”, “V” and “C” written using UVC paint in room light. (**g–i**) UVC images of the disc letters in darkness. (**j–l**) UVC images of the paint letters in darkness. The samples were irradiated by a 254 nm lamp for 2 min.

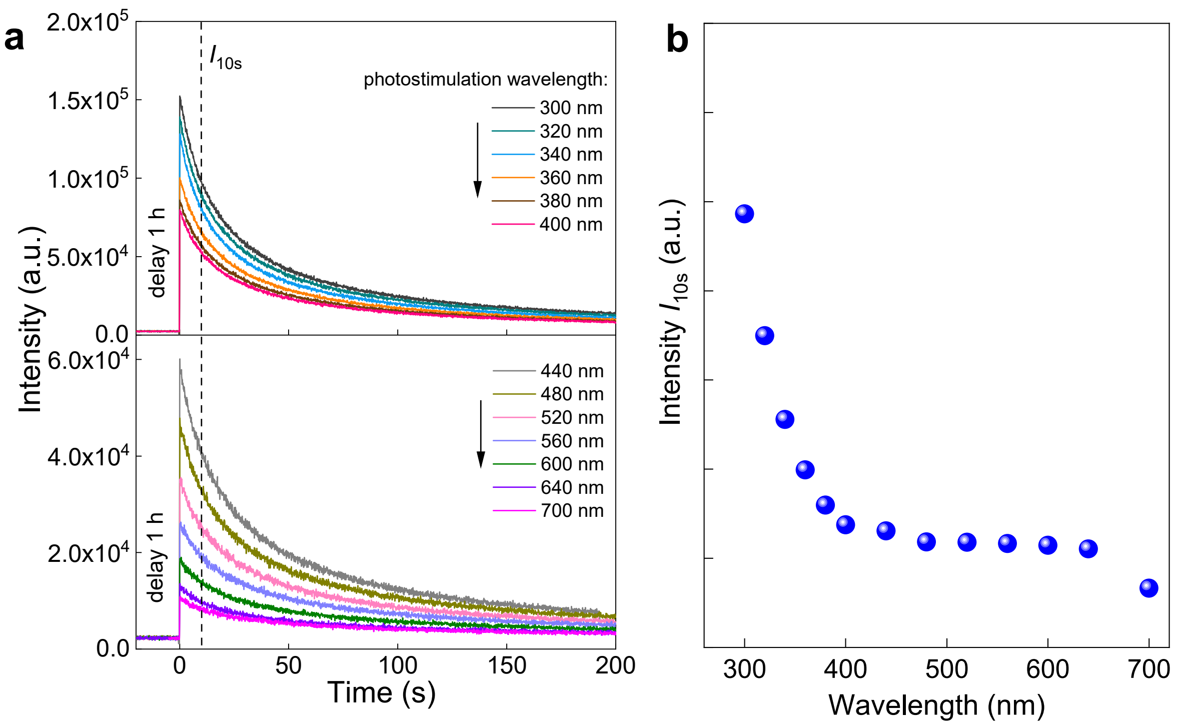

Supplementary Figure 15. PSPL in $Sr_3Y_2Si_6O_{18}$:Pr^{3+} persistent phosphor. (**a**) PSPL decay curves of a 1 h decayed disc (in darkness; pre-irradiated by a 254 nm lamp for 2 min) after 300–700 nm monochromatic light stimulation for 30 s. The monitoring wavelength is 265 nm. The PSPL intensities at time of 10 s after the stoppage of each stimulation (I_{10s}) were recorded. (**b**) Plot of PSPL intensity (I_{10s}) as a function of stimulation wavelengths. The PSPL intensity increases as the energy of the stimulation light increase. Source data are provided as a Source Data file.

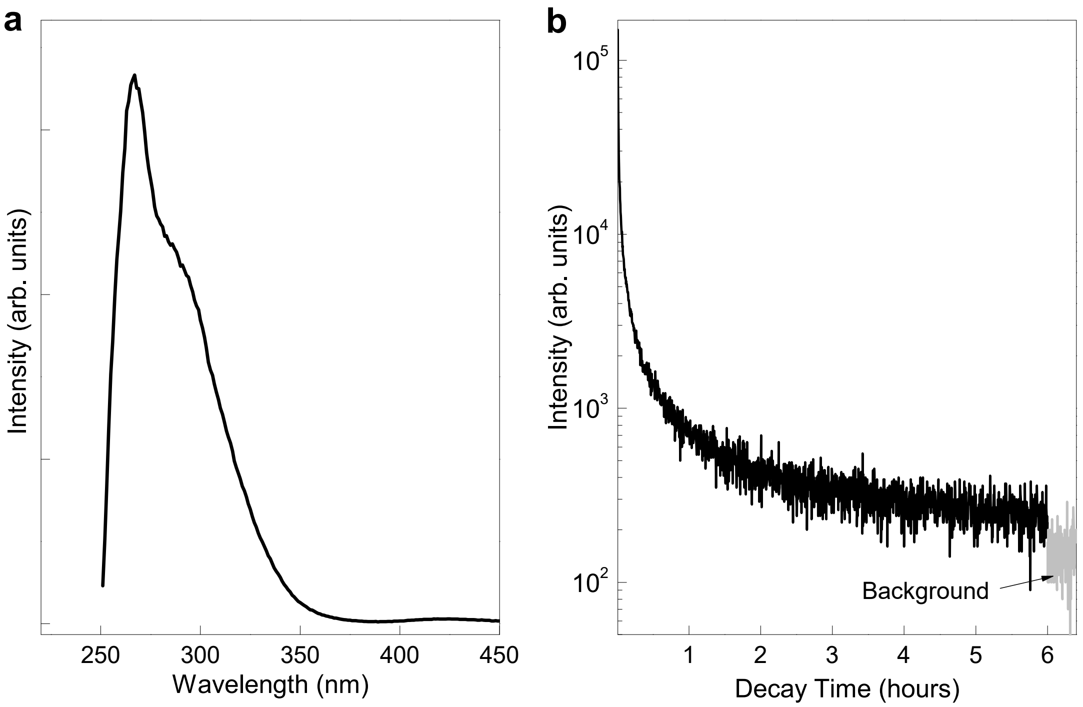

Supplementary Figure 16. Photoluminescence and persistent luminescence of silicate garnet $Ca_3Al_2Si_3O_{12}:Pr^{3+}$ UVC persistent phosphor at room temperature. (**a**) Photoluminescence emission spectrum under 220 nm excitation. The emission band peaks at 267 nm. (**b**) Persistent luminescence decay curve monitored at 267 nm after the sample was pre-irradiated by a 254 nm lamp for 2 min. Source data are provided as a Source Data file.

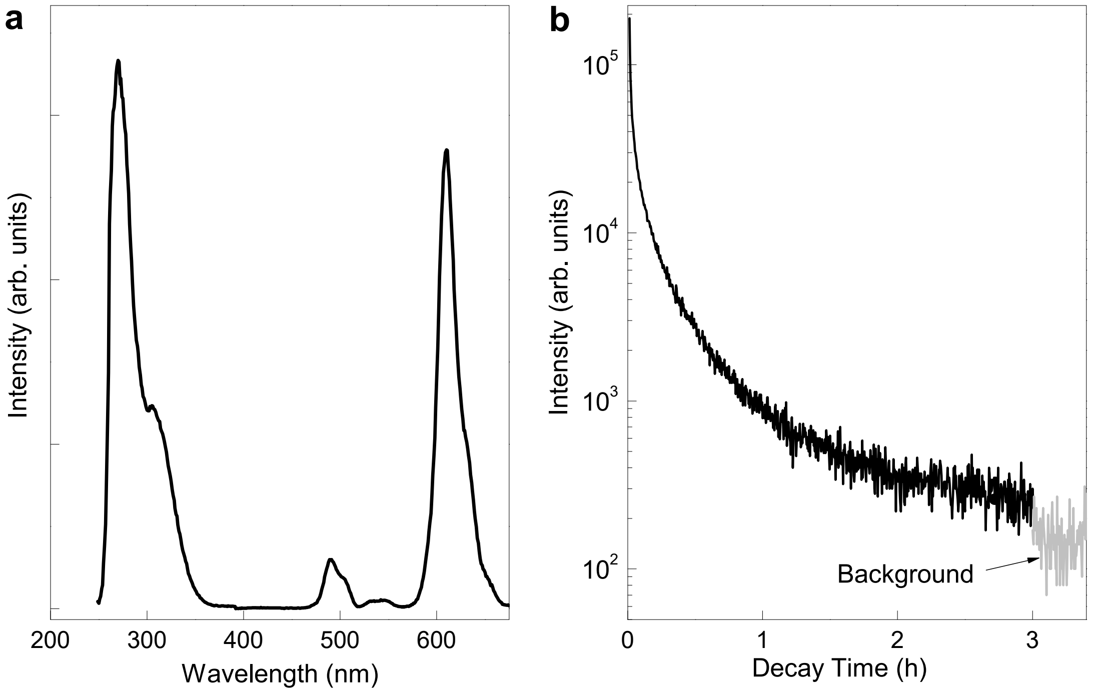

Supplementary Figure 17. Photoluminescence and persistent luminescence of oxyorthosilicate $Lu_2SiO_5:Pr^{3+}$ UVC persistent phosphor at room temperature. (**a**) Photoluminescence emission spectrum under 220 nm excitation. The emission band peaks at 270 nm. Besides the UVC emission, visible light emission bands (peaking at 490 nm and 608 nm) were also observed. (**b**) Persistent luminescence decay curve monitored at 270 nm after the sample was pre-irradiated by a 254 nm lamp for 2 min. Source data are provided as a Source Data file.

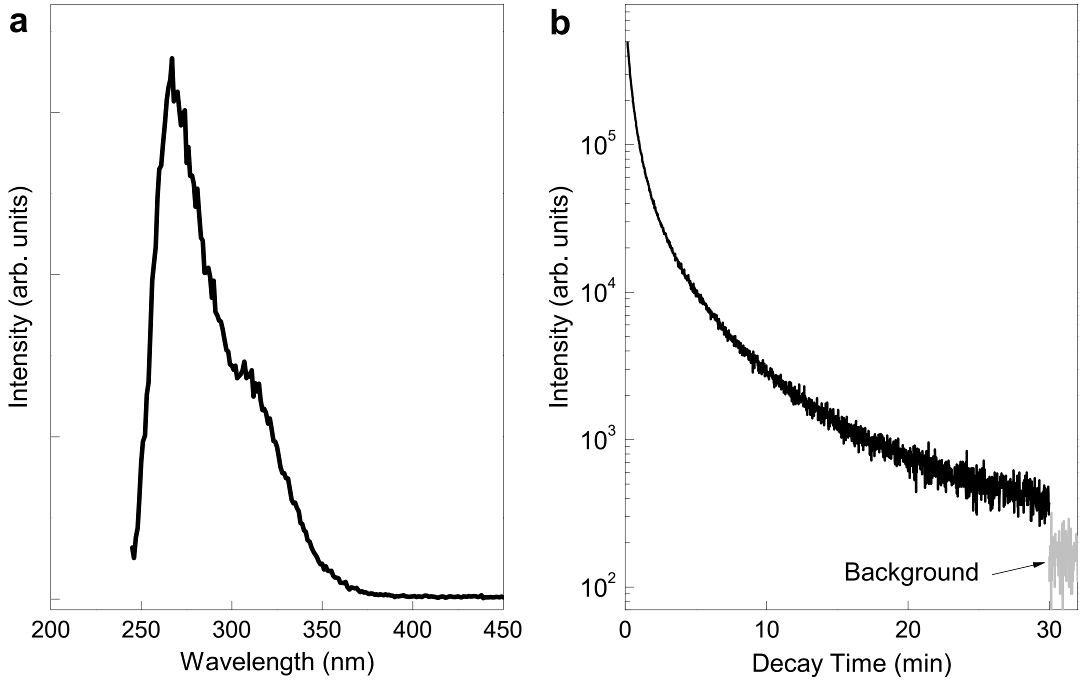

Supplementary Figure 18. Photoluminescence and persistent luminescence of orthosilicate $LiYSiO_4:Pr^{3+}$ UVC persistent phosphor at room temperature. (**a**) Photoluminescence emission spectrum under 220 nm excitation. The emission band peaks at 267 nm. (**b**) Persistent luminescence decay curve monitored at 267 nm after the sample was pre-irradiated by a 254 nm lamp for 2 min. Source data are provided as a Source Data file.

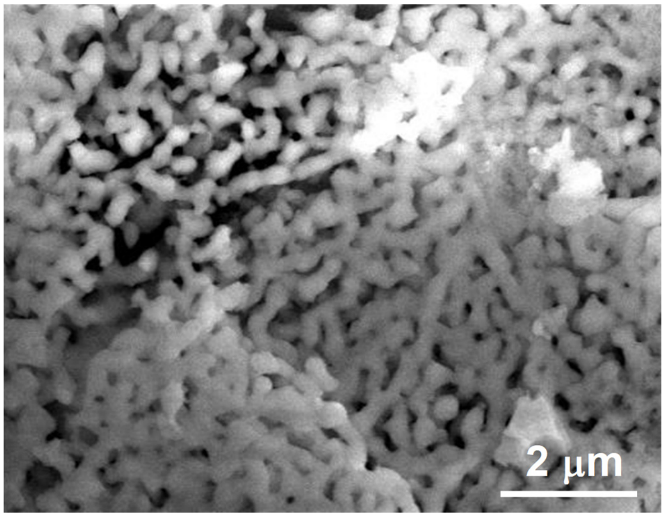

Supplementary Figure 19. Scanning electron microscope image of $Sr_3Y_2Si_6O_{18}$:Pr^{3+} UVC persistent luminescence nanoparticles. The nanoparticles were synthesized using a combustion method. The sizes of the nanoparticles are in the range of 100–200 nm.

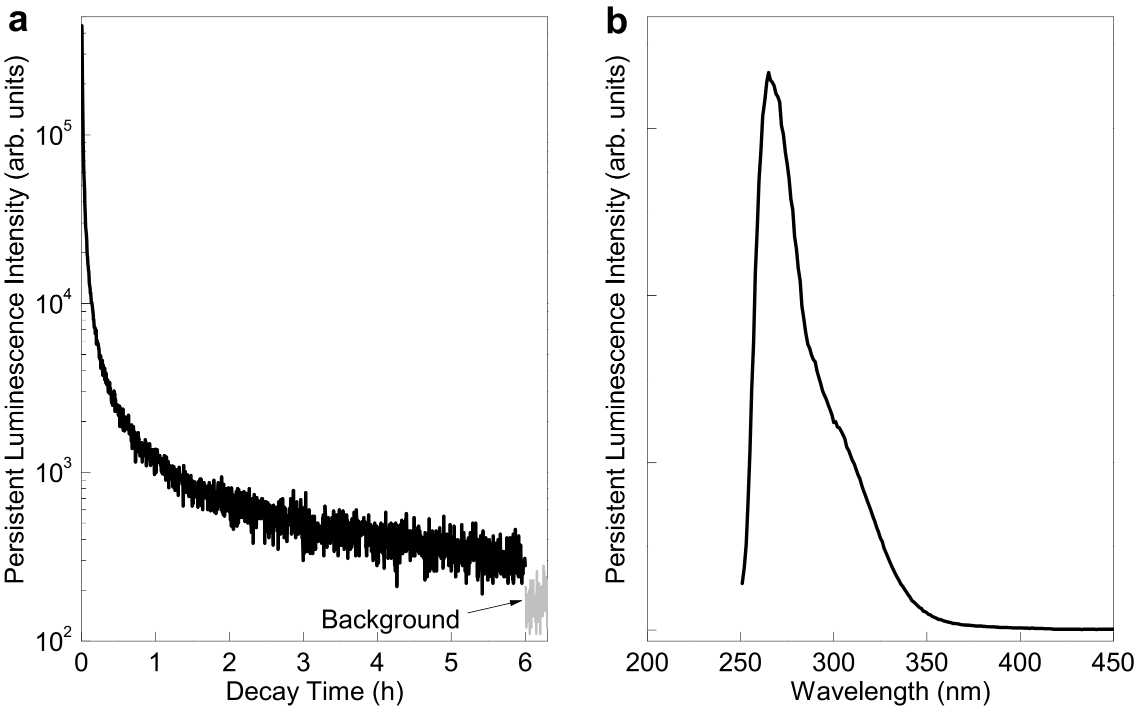

Supplementary Figure 20. Persistent luminescence of $Sr_3Y_2Si_6O_{18}:Pr^{3+}$ UVC persistent nanoparticles at room temperature. (**a**) Persistent luminescence decay curve monitored at 265 nm after the sample was pre-irradiated by a 254 nm lamp for 2 min. (**b**) Persistent luminescence emission spectrum acquired at 1 h decay. Source data are provided as a Source Data file.
